# Geolocalization of Unmanned Aerial Vehicle Images and Mapping onto Satellite Images Utilizing 3D Gaussian Splatting

**DOI:** 10.3390/s26041322

**Published:** 2026-02-18

**Authors:** Satoshi Arakawa, Kaiyu Suzuki, Tomofumi Matsuzawa

**Affiliations:** 1 Department of Information Sciences, Tokyo University of Science, Yamazaki, Noda 278-8510, Japan; matsuzawa@rs.tus.ac.jp; 2 Department of Electrical Engineering, Tokyo University of Science, Yamazaki, Noda 278-8510, Japan; kaiyu.suzuki@rs.tus.ac.jp

**Keywords:** geolocalization, UAV, 3D Gaussian Splatting, image matching

## Abstract

Geolocalization of images captured by unmanned aerial vehicles (UAVs) remains a significant challenge in Global Navigation Satellite System-denied environments. Although geolocalization is typically achieved by matching UAV images with satellite images, the viewpoint discrepancy between oblique UAV and nadir satellite images complicates this task. In this study, we employ 3D Gaussian Splatting (3DGS) to generate images from viewpoints close to the satellite viewpoint based on multiview UAV images. Assuming that the approximate flight area of the UAV is known, we propose a geolocalization method that directly establishes correspondences between 3DGS-rendered and satellite images using pixel-level image matching. These satellite images, which we refer to as wide-area satellite images, cover a larger area than the UAV observation range. Experimental results demonstrate that the proposed method achieves higher geolocalization accuracy than existing approaches that divide wide-area satellite images and perform image retrieval. Moreover, we demonstrate the potential for geographically consistent integration of independently captured and trained 3DGS models by leveraging the correspondences between 3DGS-rendered and wide-area satellite images.

## 1. Introduction

Recently, the use of unmanned aerial vehicles (UAVs) has become widespread across various fields [1,2,3], and accurate localization of UAVs is essential for precise operation [4]. The position of UAVs is primarily determined based on Global Navigation Satellite Systems (GNSS), including the Global Positioning System. However, in urban areas or mountainous regions, GNSS signals may become unreliable owing to occlusions caused by buildings or natural terrain. In such GNSS-denied environments, it becomes crucial to accurately estimate geographic location from images. Accordingly, extensive research has been conducted on image-based geolocalization, such as cross-view geolocalization (CVGL) [5], visual place recognition (VPR) [6], and absolute visual localization (AVL) [7].

CVGL and VPR typically treat images captured at unknown locations as queries and retrieve images corresponding to the capture location of the queries from a database of partitioned satellite images. Their performance is commonly evaluated using Recall@K. Representative image retrieval methods [8,9] are based on NetVLAD [10] and DINOv2 [11], while other approaches improve performance through carefully designed training and sampling strategies [12,13]. Furthermore, reranking strategies have been proposed in which image matching is performed between a query image and the top-K retrieved satellite images, and the results are reordered based on the number of matched inlier correspondences [14,15]. AVL estimates the latitude, longitude, and orientation of UAVs by comparing query images with satellite images. Existing approaches rely on image matching [16,17] or image segmentation [18,19] among others.

In image-based geolocalization tasks, the viewpoint discrepancy between the query image and satellite images complicates correspondence matching when the query image is not captured from a nadir viewpoint. Consequently, several methods have been proposed that apply a perspective transformation to oblique UAV images and use generative adversarial networks [20] to generate nadir-view images [21]. In addition, studies have reported approaches that employ diffusion models [22] to generate images with viewpoints aligned to those of satellite images [23]. However, it has been highlighted that these image generation-based methods may produce images that differ from the actual scene [24].

By contrast, Neural Radiance Fields (NeRF) [25] and 3D Gaussian Splatting (3DGS) [26] generate high-quality and photorealistic novel-view images from arbitrary viewpoints using multiview images. NeRF produces high-quality renderings, but training and rendering are generally time-consuming. 3DGS enables faster training and real-time rendering than NeRF.

The objective of this study is to improve image-based geolocalization by combining 3DGS, which generates novel-view images from multiview observations, with pixel-level image matching. Real-time capability is also crucial in geolocalization. Therefore, in this study, we employ 3DGS to generate query images that integrate multiview information from obliquely captured UAV images and to closely approximate the viewpoint of satellite images. We then establish correspondences between the 3DGS-rendered and satellite images using image-matching techniques. The proposed method achieves more robust geolocalization even under challenging conditions by relying on local correspondences. Furthermore, leveraging the obtained correspondences, we map multiple 3DGS models constructed at different locations onto a single satellite image.

## 2. Related Works

### 2.1. Pixel-Level Image Matching

Image matching aims to establish consistent spatial correspondences between images of the same scene captured at different times or from varying viewpoints [27]. Traditional image matching methods [28,29,30] establish correspondences by detecting keypoints in each image and computing feature descriptors for each keypoint. However, the handcrafted feature descriptors used in these methods have limited representational capacity.

Consequently, learning-based methods such as SuperPoint [31] and DISK [32] perform both keypoint detection and feature description. In addition, SuperGlue [33] and LightGlue [34], which introduce Transformer architectures [35] for descriptor matching, achieve highly accurate sparse matching. These keypoint-based methods, however, may fail to establish correct correspondences when keypoints are not detected in both images. Consequently, detector-free image matching methods that do not rely on keypoint detection have been proposed, and LoFTR [36] performs semidense matching. RoMa [37] performs dense matching and exhibits strong robustness to large viewpoint changes. More recent studies have further advanced image matching methods. MatchAnything [38] is designed to achieve strong generalization performance even on cross-modality image pairs not included in the training data while RoMa v2 [39] improves both the performance and computational efficiency of RoMa.

Although primarily developed for learning-based 3D reconstruction methods, DUSt3R [40] and MASt3R [41] can also establish image correspondences by directly regressing 3D point maps from image pairs.

### 2.2. Geolocalization Using Image Matching

Yang et al. [42] performed geolocalization by matching UAV images with satellite images using LoFTR. Their method focused on UAV images captured from a nadir viewpoint, assuming that the approximate flight area of the UAV and its yaw angle were known. As image rotation affects matching accuracy, the UAV images were rotated based on the yaw angle such that their orientation was aligned with north-up satellite images. The satellite images were then partitioned into patches at a scale comparable to that of the UAV images, and correspondences were established between the UAV and partitioned satellite images.

Ye et al. [15] observed that numerous studies on AVL [16,42,43,44] focused on UAV images captured from a nadir viewpoint and did not generalize to low-altitude, oblique multiview observation scenarios. For UAV images captured under such conditions, they proposed a coarse-to-fine framework that first estimated the approximate location using image retrieval and then established correspondences using image matching. Their method assumed that the UAV altitude, yaw, and pitch angles were known. Based on these prior information, the satellite images were rotated and partitioned to a scale comparable to that of the UAV images. When the top-1 retrieved satellite image did not correspond to the true location, subsequent image matching failed. Therefore, image matching was performed on the top-K retrieved satellite images. Through comparisons of multiple image retrieval and image matching methods, they concluded that the combination of CAMP [13] for image retrieval and RoMa for image matching achieved the best performance and adopted this combination as a baseline.

### 2.3. Geolocalization Using 3DGS

As 3DGS can render high-fidelity images from arbitrary viewpoints, Jiang et al. [45] and Jun et al. [46] performed image-based localization by matching query images with preconstructed Gaussian maps using 3DGS. However, these methods require maintaining and updating Gaussian maps, which leads to high memory consumption and computational cost.

By contrast, Li et al. [24] proposed a method that leverages 3DGS not as a reference map but as a tool for query image generation. This method reconstructed a scene from multiview UAV images using 3DGS and rendered images that closely resemble satellite views. Global features were then extracted from the rendered images and partitioned satellite images using DINOv2 and generalized mean pooling [47], and the top-K satellite image candidates were retrieved based on cosine similarity. Subsequently, the camera position and orientation of the rendered images were iteratively updated such that the rendered images progressively aligned with the corresponding satellite images. The satellite image with the highest cosine similarity to the updated rendered image was selected as the final geolocalization result.

Ju et al. [48] proposed a method that generated bird’s eye view image sequences from UAV videos using 3DGS and performed geolocalization using a pretrained image retrieval model. Their approach incorporated diffusion-based hard negative generation to enhance feature learning.

These existing methods reported failure cases caused by appearance changes over time and the presence of visually similar buildings located at different places.

## 3. Proposed Method

### 3.1. Datasets

**Figure 1:**
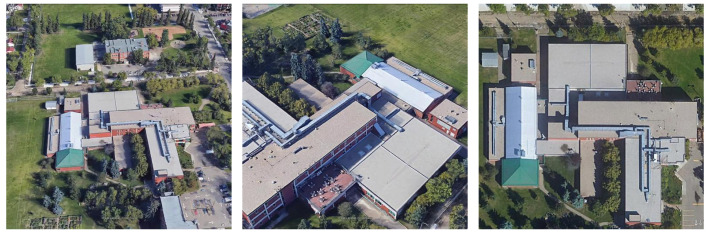
Example of UAV images (**left**, **middle**) and the corresponding satellite image (**right**) from the University-1652 dataset.

Representative datasets used for geolocalization with oblique and multiview UAV images include University-1652 [49] and SUES-200 [50]. These datasets comprise multiview UAV images and corresponding satellite images, as shown in Figure 1.

In this study, we perform geolocalization using unpartitioned satellite images that cover a larger area than the UAV observation range. We refer to these images as wide-area satellite images. The University-1652 dataset, which provides the latitude and longitude of the locations captured by each UAV, was used to obtain wide-area satellite images. The University-1652 dataset covers 1652 buildings across 72 universities worldwide and was acquired using 3D models provided by Google Earth. The UAV images were captured while the UAV flew three loops around the target building along a spiral trajectory, gradually decreasing its altitude from 256 to 121.5 m. A total of 54 UAV images are provided for each building. The dataset is divided into training data for model learning and test data for performance evaluation.

**Figure 2:**
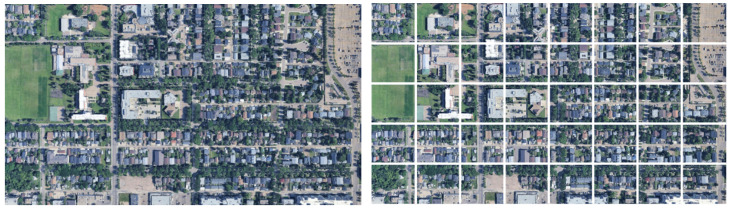
Example of a wide-area satellite image (**left**) and the 8×5 tiled image (**right**).

Wide-area satellite images were obtained from Google Earth Pro with an image size of 1280×720 pixels and a spatial resolution of approximately 0.7m per pixel. An example of the acquired wide-area satellite images is shown in Figure 2 (left). In geolocalization, when satellite images are partitioned, the accuracy decreases if the ground-truth location of the query image is on a tile boundary [6]. In this study, when acquiring wide-area satellite images, we ensured that the ground-truth location of each query image was contained within a single tile by partitioning the image into an 8×5 grid, as shown in Figure 2 (right). This setting avoids the accuracy degradation caused by partitioning wide-area satellite images in existing approaches.

### 3.2. GeoLocalization

**Figure 3:**
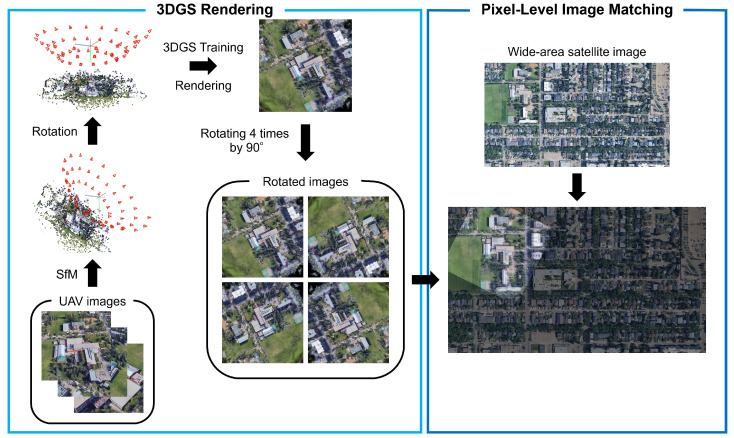
Overview of the proposed method.

In this study, we assume a scenario in which the approximate flight area of the UAV is known, whereas its altitude, yaw, and pitch angles are unknown. An overview of the proposed method is illustrated in Figure 3.

We first use COLMAP [51], a structure-from-motion method, to estimate the camera positions and orientations of multiview UAV images and to obtain a point cloud of the reconstructed scene. We then detect a plane (ground plane) from the point cloud using RANSAC [52], following Li et al. [24]. We rotate the camera poses and the point cloud such that the normal vector of the detected ground plane aligns with the vertical axis of the 3D coordinate system. Subsequently, we train a 3DGS model and render images from viewpoints close to the satellite viewpoint along this axis. The rendered images have a resolution of 384×384 pixels.

As the UAV yaw angle is unknown in this study, the top of a 3DGS-rendered image is not necessarily oriented northward, unlike the wide-area satellite image. To reduce the impact of rotation on image matching, we rotate each rendered image by 90° increments and perform image matching with the wide-area satellite image four times, once for each rotation. We then estimate a similarity transformation matrix from the resulting correspondences and select the transformation with the largest number of inliers as the final result. For estimating the similarity transformation matrix, we use OpenCV’s estimateAffinePartial2D.

## 4. Experiments and Results

In this study, we compared image matching methods and evaluated the proposed method against existing approaches. All experiments were conducted on a machine equipped with an Intel Core i9-9900K CPU and an NVIDIA GeForce RTX 4070 GPU.

### 4.1. Evaluation of the Proposed Method

As shown in Figure 3, we used the estimated similarity transformation matrix to overlay the 3DGS-rendered image onto the wide-area satellite image. We then evaluated the results based on (i) the tile index containing the center of the rendered image after partitioning the wide-area satellite image into 8×5 tiles, (ii) the rotation angle, and (iii) the overlapping ratio between the rendered image and the wide-area satellite image. Accordingly, we used Accuracy as the evaluation metric.

For query *i*, we defined the ground-truth tile location containing the center of the rendered image as $p_{i}=\left(x_{i},\;y_{i}\right)\;\left(x_{i}\in \left\{0,1,\ldots ,7\right\},\;y_{i}\in \left\{0,1,\ldots ,4\right\}\right)$, the ground-truth rotation angle as $\theta_{i}\in \left[0,360\right)$, and the ground-truth overlapping ratio between the rendered image and the wide-area satellite image as $S_{i}\in \left(0,1\right]$. The corresponding values computed from the estimated similarity transformation matrix were denoted by ${\hat{p}}_{i}=\left({\hat{x}}_{i},\;{\hat{y}}_{i}\right)$, ${\hat{\theta }}_{i}$, ${\hat{S}}_{i}$, respectively. The center-to-center distance between adjacent tiles was 160 pixels in the *x*-direction and 144 pixels in the *y*-direction. Given the spatial resolution of 0.7m per pixel, the tile-position error $e_{i}^{\mathrm{pos}}$ is defined in Equation (1). The rotation error $e_{i}^{\mathrm{ang}}$ and the relative area error $e_{i}^{\mathrm{area}}$ are defined in Equations (2) and (3), respectively.

(1) $$e_{i}^{\mathrm{pos}}=\sqrt{{\left(\left({\hat{x}}_{i}-x_{i}\right)\cdot 160\right)}^{2}+{\left(\left({\hat{y}}_{i}-y_{i}\right)\cdot 144\right)}^{2}}\cdot 0.7,$$

(2) $$e_{i}^{\mathrm{ang}}=\min\left(|{\hat{\theta }}_{i}-\theta_{i}\left|,\;360-\right|{\hat{\theta }}_{i}-\theta_{i}|\right),$$

(3) $$e_{i}^{\mathrm{area}}=\left|\frac{{\hat{S}}_{i}}{S_{i}}-1\right|.$$

In this study, a query was regarded as correct if it satisfied $e_{i}^{\mathrm{pos}}=0$, $e_{i}^{\mathrm{ang}}<3{}^{\circ}$, and $e_{i}^{\mathrm{area}}<0.3$. Here, the threshold 0.3 for $e_{i}^{\mathrm{area}}$ was introduced to tolerate an error of approximately 15% in the horizontal and vertical dimensions of the rendered image. The correctness indicator $\delta_{i}$ for query *i* was defined as in Equation (4).

(4) $$\delta_{i}=\left\{\begin{array}{cc} 1 & \mathrm{if}\;\left(e_{i}^{\mathrm{pos}}=0\right)\wedge \left(e_{i}^{\mathrm{ang}}<3{}^{\circ}\right)\wedge \left(e_{i}^{\mathrm{area}}<0.3\right),\\ 0 & \mathrm{otherwise}. \end{array}\right.$$

Letting *N* denote the total number of queries, accuracy was defined as in Equation (5).

(5) $$\mathrm{Accuracy}=\frac{1}{N}\sum_{i=1}^{N}\delta_{i}.$$

### 4.2. Comparison of Image Matching Methods

Some 3DGS-rendered images were rotated relative to the wide-area satellite images because the UAV yaw angle is unknown. We compared the accuracy of several image matching methods under different rotation angles to evaluate how the rotation angle of rendered images affected matching performance.

**Figure 4:**
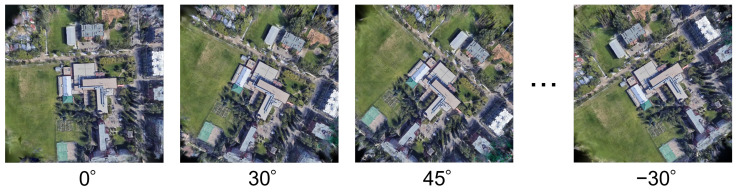
Rotated 3DGS-rendered images.

In the test data of University-1652, we observed that the first UAV image was captured facing north in most cases, except for sequence 0001. We defined the orientation of the first UAV image as 0°, that is, the nonrotated state with respect to the wide-area satellite image. We then generated rendered images rotated by ±30k°(k=1,2,…,6) and by ±45°. An example of the first UAV image is shown in Figure 1 (left), and examples of rotated rendered images are shown in Figure 4.

**Figure 5:**
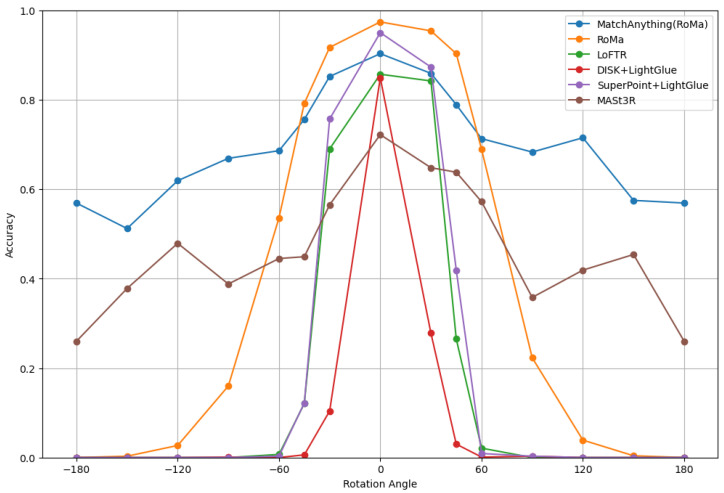
Geolocalization accuracy under different rotation angles of rendered images.

We performed image matching between the rotated rendered and wide-area satellite images. The evaluated matchers were MatchAnything (RoMa), RoMa, LoFTR, DISK + LightGlue, SuperPoint + LightGlue, and MASt3R. The results are shown in Figure 5.

As illustrated in Figure 5, the accuracy decreased as the rotation angle increased, indicating that rotation of the rendered images affected the matching performance with wide-area satellite images. Among the evaluated image matching methods, RoMa achieved the highest accuracy for rotation angles ≤45°, whereas MatchAnything (RoMa) performed best for rotation angles ≥60°. Performing image matching multiple times using rendered images rotated by fixed angles could reduce the impact of unknown rotation relative to the wide-area satellite image. However, geolocalization accuracy and computational cost, that is, the number of matching attempts, have a trade-off relationship. Based on these results, we adopted RoMa and performed image matching four times by rotating each rendered image in 90° increments. This strategy reduced the maximum residual rotation to 45°. In the following experiments, we used RoMa as the image matching method and compared the proposed method with existing approaches.

### 4.3. Comparison with Existing Approaches

As methods for geolocalization based on a single query image, we compared the proposed method with CAMP, the baseline method reported by Ye et al. [15], and the method proposed by Li et al. [24]. As evaluation metrics, we used Accuracy and SDM@1 based on the Spatial Distance Metric (SDM) [43]. We also measured the computational time.

The accuracy of the proposed method was evaluated based on Equation (5). By contrast, for existing retrieval-based methods, the task was to rank the correct tile among the partitioned tiles of the wide-area satellite image. Accordingly, for the existing methods, we reported Recall@1, which is the fraction of queries for which the top-1 retrieved result corresponds to the ground-truth tile, as accuracy. Moreover, letting *N* denote the total number of queries, SDM@1 was defined using the position error $e_{i}^{\mathrm{pos}}$ in Equation (1), as shown in Equation (6). Whereas Recall@1 is a discrete metric that takes values of 0 or 1, SDM@1 is a continuous metric distributed between 0 and 1 that accounts for spatial proximity. The scaling coefficient *s* for the position error $e_{i}^{\mathrm{pos}}$ was set such that Equation (7) held when $|{\hat{x}}_{i}-x_{i}|=1$ and $|{\hat{y}}_{i}-y_{i}|=1$ in Equation (1).

(6) $$\mathrm{SDM}@1=\frac{1}{N}\sum_{i=1}^{N}\frac{1}{\exp\;\left(s\cdot e_{i}^{\mathrm{pos}}\right)},$$

(7) $$\frac{1}{\exp\;\left(s\cdot \sqrt{160^{2}+144^{2}}\cdot 0.7\right)}=\frac{1}{3}.$$

Ye et al. [15] first rotated the satellite image according to the UAV yaw angle and then partitioned the satellite image based on the UAV altitude and pitch angle, as well as the camera field of view, such that each tile was at approximately the same scale as the UAV image. In our experiment, we used a north-facing UAV image (Figure 1, left) as the query image and partitioned the wide-area satellite image by setting the UAV altitude to 256 m [49] and the pitch angle to 45° [48]. The camera field of view was computed from the camera parameters estimated by COLMAP. We then partitioned the wide-area satellite image by centering each crop at the tiles of the 8×5 grid.

Li et al. [24] iteratively updated the camera position and orientation for rendering 3DGS images; however, their implementation code was not publicly available. Although the paper reported failure cases in the camera update process, we assumed that all updates succeeded. Therefore, we used the 0° rendered image (Figure 4, left) as the query image.

Following both papers, we set the image resolution to 384×384 pixels. The results of each method are summarized in Table 1. In addition to existing approaches and the proposed method, we also reported the result of directly matching the north-facing UAV image with the wide-area satellite image using RoMa to verify the effectiveness of 3DGS in reducing the viewpoint discrepancy between the query and wide-area satellite images. For the UAV + RoMa method, the tile was selected based on the center of the UAV image. In Table 1, a check mark indicates that the wide-area satellite image is partitioned into tiles, whereas a cross indicates that it is not. The best results are highlighted in bold.

As summarized in Table 1, the proposed method achieved the highest accuracy despite not partitioning the wide-area satellite image. It also achieved an accuracy comparable to that of CAMP Top5 + RoMa, which assumed partitioning of the wide-area satellite image. The DINOv2-based method achieved a lower accuracy than those of the other methods; however, it achieved Recall@5 of 0.912, indicating strong retrieval performance.

CAMP Top5 + RoMa achieved the highest SDM@1. This was because, when the proposed method failed in geolocalization, the center of the rendered image often fell into a tile far from the ground-truth tile or even outside the wide-area satellite image, whereas the existing methods tended to select tiles closer to the ground-truth tile. However, the proposed method did not use the UAV yaw angle. If the yaw angle were known, the matching could be performed using the 0° rendered image shown on the left of Figure 4 and the wide-area satellite image. In this setting, as shown in Figure 5, the proposed method would achieve the accuracy of 0.974 and SDM@1 of 0.979, with a runtime of 2.04 s.

**Figure 6:**
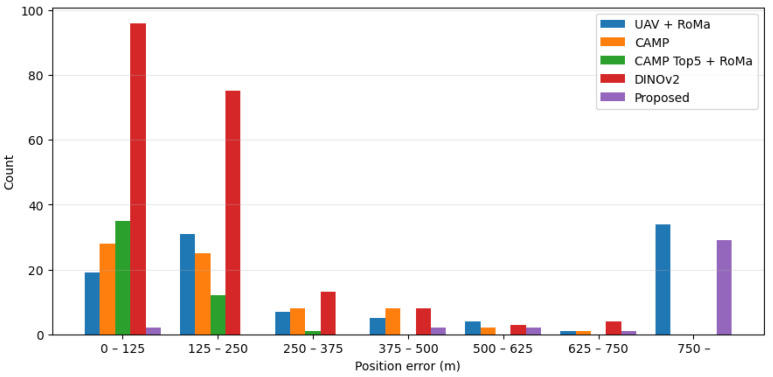
Distribution of position errors for failed cases.

**Figure 7:**
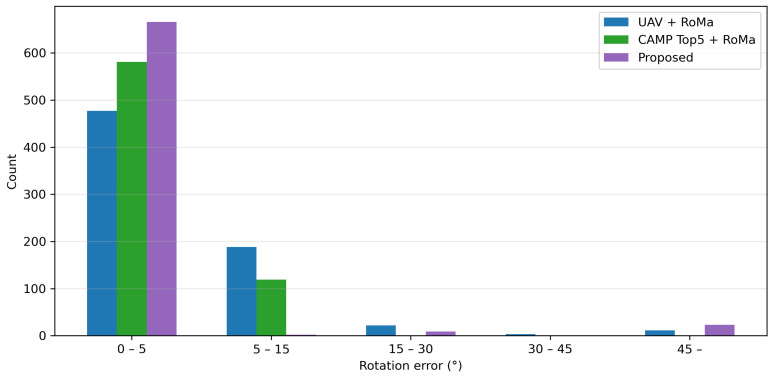
Distribution of rotation errors for methods using RoMa.

Figure 6 shows the distribution of position errors for failed geolocalization cases ($e_{i}^{\mathrm{pos}}\;>\;0$) because successful cases had $e_{i}^{\mathrm{pos}}=0$. Figure 7 shows the distribution of rotation errors $e_{i}^{\mathrm{ang}}$. The rotation error was not defined for the CAMP and DINOv2-based method because they performed image retrieval from partitioned satellite images. Therefore, we reported the rotation errors only for the methods that perform the pixel-level image matching with the satellite image. The vertical axis in Figure 6 and Figure 7 indicates the number of cases.

As shown in Figure 6 and Figure 7, when the proposed method failed in geolocalization, its errors tended to be larger than those of the other methods. We considered that this was because the wide-area satellite image was not partitioned in the proposed method.

## 5. Discussion

### 5.1. Appearance Changes over Time

**Figure 8:**
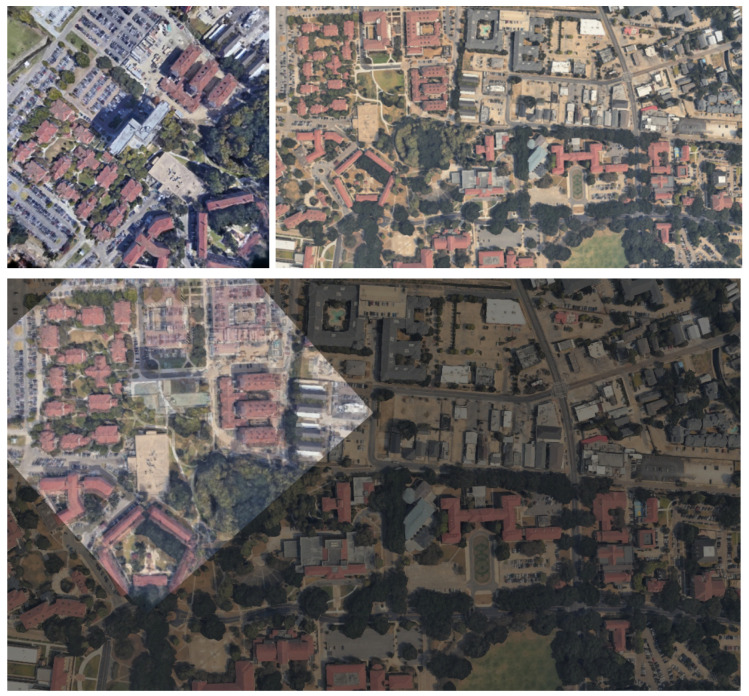
Correspondence result between a 3DGS-rendered image and the wide-area satellite image with partial appearance changes.

The proposed method successfully established correct correspondences even for data in which partial appearance differences exist between the 3DGS-rendered and wide-area satellite images due to temporal changes. Figure 8 shows a geolocalization result for such a case. In Figure 8, the top row shows the 3DGS-rendered image and the wide-area satellite image, and the bottom row shows the matching result between them. In the wide-area satellite image, a newly constructed building is visible near the upper-left region of the rendered image, and a building near the center is no longer present.

**Figure 9:**
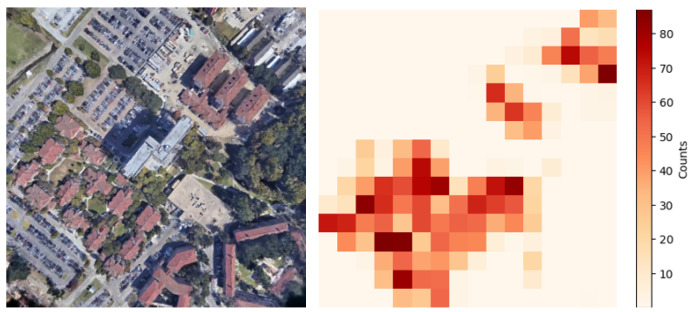
Distribution of inliers in the rendered image.

Figure 9 shows a heatmap visualizing the distribution of inliers in the rendered image. As shown in the heatmap, the correspondences are primarily established in regions where the appearance has not changed.

### 5.2. Visually Similar Buildings at Different Locations

**Figure 10:**
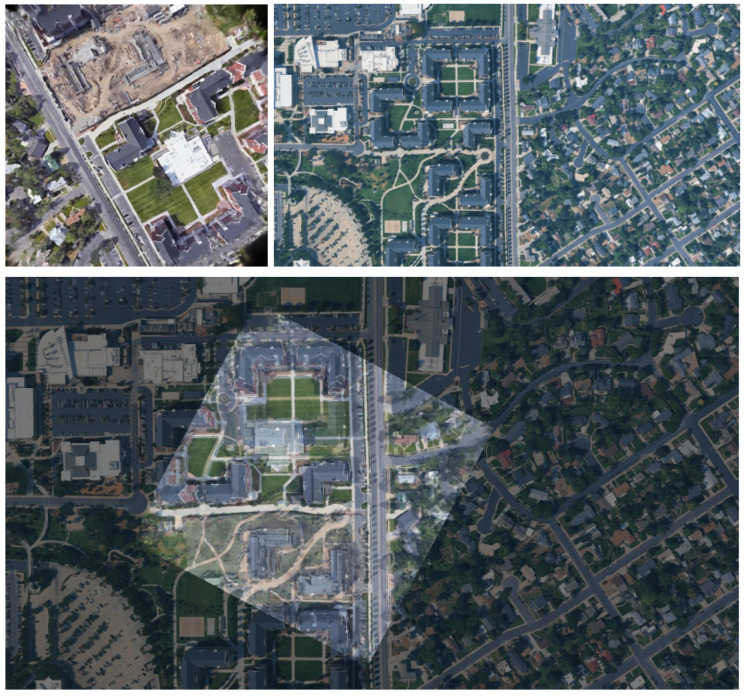
Correspondence results between a 3DGS-rendered image and a wide-area satellite image with multiple visually similar buildings.

The proposed method successfully established correct correspondences even for data in which multiple visually similar buildings exist in a wide-area satellite image. Figure 10 shows a geolocalization result for such a case. In Figure 10, the top row shows the 3DGS-rendered image and the wide-area satellite image, and the bottom row image shows the matching result between them. In the wide-area satellite image, several buildings with shapes similar to the L-shaped building located near the center of the rendered image are distributed. In addition, owing to temporal appearance changes, newly constructed buildings are visible near the upper-left and central regions of the rendered image, and a white building originally located near the center is no longer present. These factors further increase the difficulty of geolocalization.

**Figure 11:**
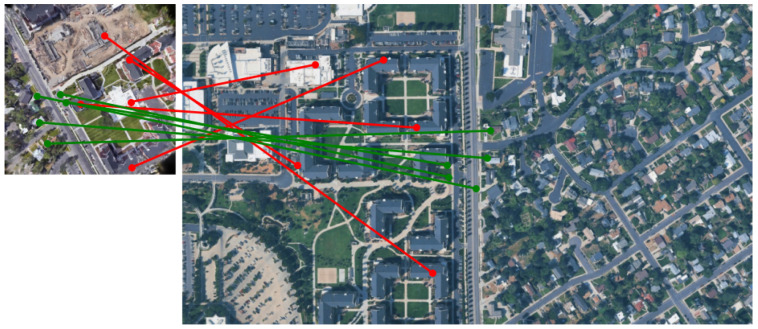
Correct correspondences (green) and incorrect correspondences (red).

Figure 11 shows the correspondence points between the rendered image and the wide-area satellite image. Correct correspondences are shown in green while incorrect correspondences are shown in red, with five points visualized for each. Owing to the presence of numerous visually similar buildings, several incorrect correspondences were observed. Nevertheless, accurate geolocalization can still be achieved based on a subset of correct correspondences.

### 5.3. Strategy for Reducing Computational Time

In this study, we considered the scenario in which the UAV yaw angle was unknown. Therefore, we performed image matching four times between the wide-area satellite image and the 3DGS-rendered images rotated in 90° increments, estimated a similarity transformation matrix from the resulting correspondences, and selected the final estimate based on the number of inliers. Although this procedure improved geolocalization accuracy, it increased the computational time because image matching was executed multiple times. We investigated a strategy based on the inlier ratio of the correspondences to reduce unnecessary matching trials.

Specifically, we conducted an experiment in which the matching process was terminated early: if the inlier ratio exceeded a predefined threshold λ, the current estimate was accepted as the final result and the remaining matching trials were skipped. Table 2 reports the accuracy and the computational time for different values of λ.

**Figure 12:**
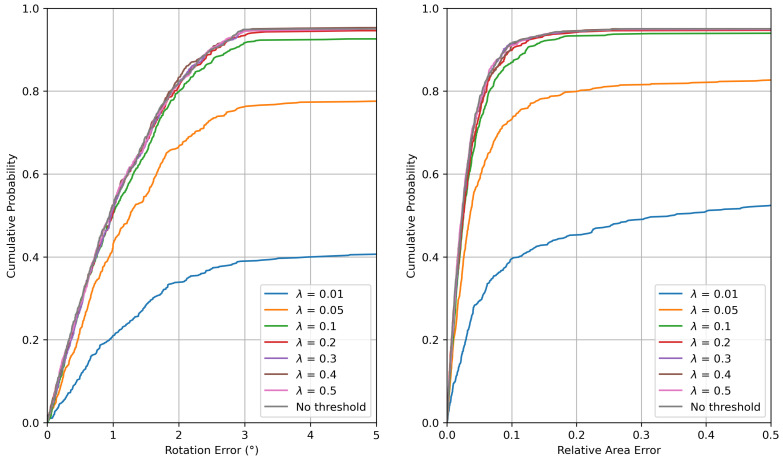
CDFs of the rotation and relative area errors.

As summarized in Table 2, increasing the inlier ratio threshold λ improved the accuracy at the cost of longer runtime. This indicated a trade-off between geolocalization accuracy and computational time, which was dominated by the number of image-matching trials. Figure 12 shows the cumulative distribution functions (CDFs) of the rotation error $e_{i}^{\mathrm{ang}}$ and the relative area error $e_{i}^{\mathrm{area}}$ when using different thresholds λ, as well as the case without thresholding.

As shown in Figure 12, larger values of λ led to smaller rotation and relative area errors. Moreover, the error distributions exhibited no substantial differences for λ≥0.2. Based on the results in Table 2 and Figure 12, setting λ=0.1 or λ=0.2 reduced unnecessary matching trials while maintaining high geolocalization accuracy. Note that these results were obtained using RoMa for image matching, and the appropriate threshold may differ for other matching methods.

### 5.4. Partitioning of Wide-Area Satellite Images

In Section 4.3, we compared the proposed method with the existing methods. The proposed method assumed a scenario where only the approximate flight area of the UAV was known, whereas the existing methods used additional UAV information to avoid underestimating their performance. In the following, we adopted the same assumption as the proposed method and conducted an experiment regarding the partitioning of wide-area satellite images for the existing methods.

**Figure 13:**
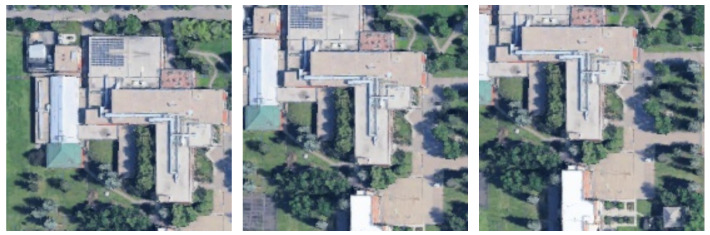
Examples of wide-area satellite image partitioning for boundary cases.

When the UAV altitude and pitch angle are unknown, it is difficult to partition the wide-area satellite images into tiles that are at approximately the same scale as the UAV images. Therefore, we partitioned the wide-area satellite images such that each tile covered an area comparable to the satellite images in the University-1652 dataset shown in Figure 1 (right). In addition, although the wide-area satellite images were acquired to ensure that the ground-truth location was contained within a single tile, we also considered cases where the ground-truth location was on a tile boundary. To evaluate such boundary cases, we generated additional partitions by shifting the partition grid by 30 and 50 pixels in both the *x* and *y* directions (offsets). Examples of the partitions are shown in Figure 13.

We performed geolocalization using CAMP for each partition, and the results in terms of Recall@1 and Recall@5 are reported in Table 3.

Introducing overlaps between adjacent tiles has been proposed to mitigate tile-boundary effects [6,42]. As summarized in Table 3, both Recall@1 and Recall@5 decreased when the ground-truth location was on a partition boundary. Therefore, for tile-based image retrieval methods, the geolocalization performance may depend on the partitioning scheme. By contrast, the proposed method can perform geolocalization without being affected by tile boundaries because it directly establishes correspondences between 3DGS-rendered and satellite images.

### 5.5. Spatial Resolution of Wide-Area Satellite Images

In this study, the wide-area satellite images were obtained from Google Earth Pro with a spatial resolution of approximately 0.7 m per pixel. We additionally acquired wide-area satellite images with a spatial resolution of approximately 1.0 m per pixel and conducted a preliminary experiment to examine the impact of spatial resolution on the proposed geolocalization method. The image size was fixed to 1280×720 pixels in both settings. For this preliminary experiment, we used 100 samples from the test data of the University-1652 dataset. Table 4 lists the accuracy and SDM@1 values of the proposed method at spatial resolutions of 0.7 and 1.0 m.

As summarized in Table 4, the performance of the proposed method degraded as the spatial resolution became coarser. Because the image size was fixed in this experiment, increasing the spatial resolution from 0.7 to 1.0 m per pixel approximately doubled the ground area covered by the wide-area satellite image. Consequently, the overlapping ratio $S_{i}$ between the rendered image and the wide-area satellite image decreased from approximately 0.2 to 0.1, making it more difficult for image matching to establish correct correspondences. These results suggested that wide-area satellite images with a spatial resolution of around 0.7 m per pixel were more suitable for the proposed method.

### 5.6. When the Approximate Flight Area of the UAV Is Unknown

In this study, we directly matched 3DGS-rendered images with a wide-area satellite image under the assumption that the approximate flight area of the UAV is known. However, when the flight area is unknown, directly matching against a wide-area satellite image becomes challenging. In such cases, retrieval-based methods such as CAMP or DINOv2 can be used to perform image retrieval from a database of partitioned satellite images. The proposed method can then be applied to the top-ranked retrieved satellite images, which we expect to enable more accurate geolocalization. Therefore, the proposed method can be extended to scenarios in which the UAV flight area is unknown by combining it with existing retrieval-based geolocalization approaches.

### 5.7. Our Pipeline

Our pipeline can be divided into two stages: (i) generation of rendered images using 3DGS and (ii) image matching with wide-area satellite images. In the following, we discuss each stage.

**Figure 14:**
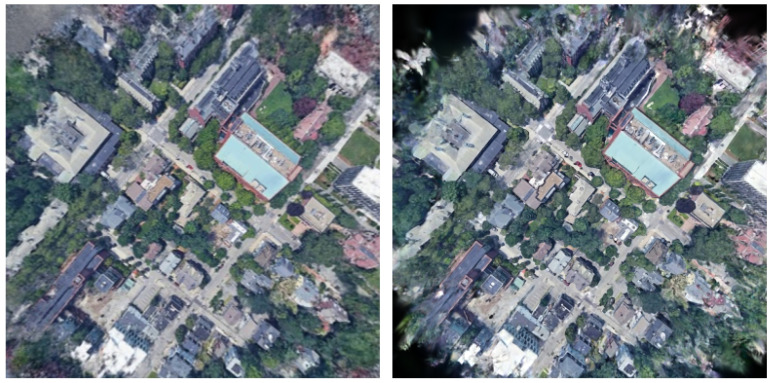
Comparison of rendered images by NeRF (**left**) and 3DGS (**right**).

First, we discuss the generation of 3DGS-rendered images. In this study, we adopted 3DGS instead of NeRF for query image generation, considering the real-time requirement of geolocalization. Using the same UAV images and camera parameters, Figure 14 compares a NeRF-rendered image (left) with a 3DGS-rendered image (right). In particular, in the upper-left region of the rendered images, the 3DGS-rendered image contains areas without 3D Gaussians, whereas the NeRF-rendered image preserves slightly more appearance details. However, the building edges appear sharper in the 3DGS-rendered image.

When real-time operation is not required, using NeRF is also a viable option. Moreover, NeRF-GS [53], which combines NeRF and 3DGS, has been proposed. Comparing how these novel-view synthesis methods affect geolocalization performance in our framework would be valuable. As future work, we will perform such comparisons and investigate the most suitable synthesis method for our geolocalization task.

Furthermore, because the University-1652 dataset was collected from Google Earth, it does not contain dynamic objects such as pedestrians or vehicles, unlike UAV images captured in real-world environments. Such dynamic objects may act as noise in 3D reconstruction and in 3DGS-rendered images. Consequently, DroneSplat [54] suppresses the influence of dynamic objects. Moreover, whereas the present study focuses on UAV images, Horizon-GS [55] and CrossView-GS [56] build 3DGS by combining UAV images with ground-level images. These methods could serve as alternatives to the rendered-image generation stage in our pipeline.

Second, we discuss image matching with wide-area satellite images. CAMP, which was used in the accuracy comparison, relies on a model trained on the University-1652 dataset. By contrast, the proposed method does not depend on a specific geolocalization dataset. Therefore, it is applicable to diverse environments beyond building-centric scenarios, and we expect it to have strong generalization capability. In addition, new image matching methods such as RoMa v2 have been proposed. As future work, we will evaluate the performance of our framework whenever new image matching methods are proposed and, if necessary, update our pipeline to a more accurate matcher.

### 5.8. Applications of 3DGS Enabled by Alignment with Wide-Area Satellite Images

Recently, large-scale 3DGS methods that reconstruct city-scale scenes, such as CityGaussian [57] and VastGaussian [58], have achieved efficient training and rendering. In addition, Skyfall-GS [59] reconstructs 3D scenes using only satellite images. However, these methods require substantial computational resources, and a sufficient set of input images may not be available in some cases.

Multiple 3DGS models that are captured and trained independently can be integrated in a geographically consistent manner by leveraging the correspondences between 3DGS-rendered and wide-area satellite images obtained in this study. This enables partial reconstruction of only the required locations within a city-scale area. The integration can be implemented by applying the wide-area satellite coordinate system to the ply files that store the optimized 3D Gaussians for each region. Specifically, we align the ground planes detected by RANSAC across regions and update the positions, rotations, and scales of the 3D Gaussians using the similarity transformation estimated by overlaying the rendered image onto the wide-area satellite image.

**Figure 15:**
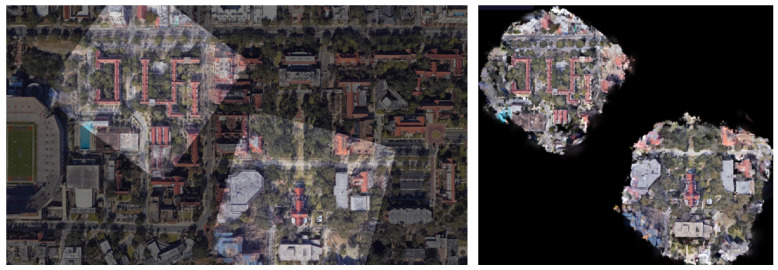
Alignment of 3DGS-rendered images with the wide-area satellite image (**left**) and an overhead rendering after integrating the corresponding 3D Gaussians (**right**).

Using the University-1652 dataset, we matched 3DGS-rendered images from two locations to a single wide-area satellite image and integrated the corresponding 3D Gaussians. In Figure 15, the left shows the alignment results of the two rendered images with the wide-area satellite image, and the right shows an overhead rendering after integrating the 3D Gaussians. This is an additional benefit of our pixel-level image matching, which aligns 3DGS-rendered images with wide-area satellite images, unlike existing 3DGS-based geolocalization methods.

**Figure 16:**
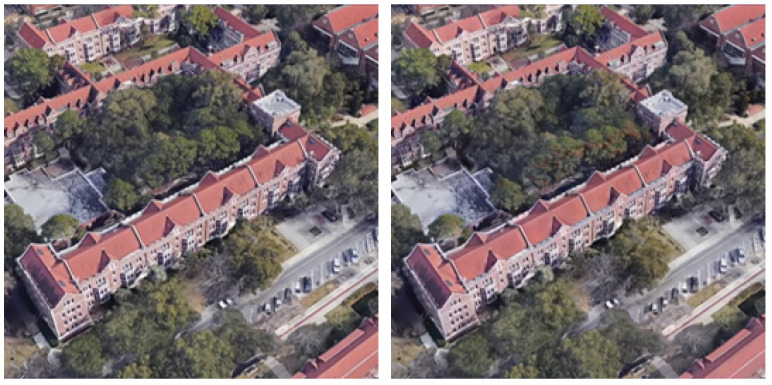
Before integration (**left**) and after integration (**right**).

However, we observed a degradation in rendering quality when the spherical harmonic parameters of the 3D Gaussians were not adjusted. Rendered images observed from the same location before and after integration are shown in Figure 16. Although slight differences are visible, we considered the quality degradation to be acceptable.

Furthermore, after transforming the 3DGS coordinate system into the wide-area satellite coordinate system, the current viewpoint, as shown in Figure 16, can be visualized on the wide-area satellite image. This is achieved by extracting the camera position and orientation from the 3DGS viewer.

### 5.9. Future Work

In this study, we demonstrated that alignment with wide-area satellite images is possible even under partial appearance changes. For future work, we will consider applications to natural disasters. In such scenarios, UAVs are highly effective owing to their convenience and mobility [60], enabling the collection of information for monitoring disaster situations, mapping affected areas, and assessing damage to roads and buildings [61]. Digital archives that record disaster situations are significant [62,63], and linking archived data with geographic location information is effective for enhancing disaster awareness and risk perception. However, some archived data do not contain location information as metadata [64]. By applying the proposed method, 3DGS models constructed from post-disaster images can be aligned with wide-area satellite images acquired before the disaster. This would enable disaster conditions to be recorded and preserved in association with geographic information, even when the data do not contain accurate location metadata. Such a capability is expected to be useful for applications including disaster-related digital archives and hazard map construction.

**Figure 17:**
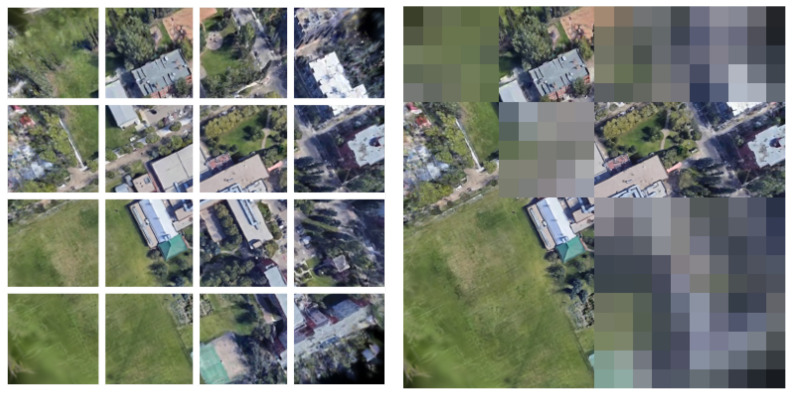
Grid partition of a rendered image (**left**) and a rendered image with blur and mosaic effects (**right**).

Accordingly, we conducted an experiment in which blur and mosaic effects were applied to parts of the 3DGS-rendered images to introduce regions that were difficult to match with wide-area satellite images. As shown in Figure 17 (left), each rendered image was divided into a 4×4 grid, and the effects were applied to 2n cells (n=1,2,…,7) out of 16 cells. An example with eight processed cells is shown in Figure 17 (right). In natural disasters, such as landslides and building damage, some areas may still remain usable as correct correspondences with wide-area satellite images. By contrast, the blur and mosaic effects in our experiment make the processed regions unusable for correspondence estimation. Therefore, this experimental setting can be regarded as an evaluation under a more challenging appearance-change condition.

The geolocalization accuracy under different proportions of blur and mosaic regions is summarized in Table 5. For each value of 2n, the processed cells were selected at random, and we reported the mean and standard deviation of accuracy over five trials.

As summarized in Table 5, the accuracy decreased as the proportion of blur and mosaic regions increased. Nevertheless, even when 50% of the rendered image comprised hard-to-match regions, the geolocalization accuracy remained 0.724 ± 0.024. This indicates that the proposed method is robust to challenging appearance changes.

**Figure 18:**
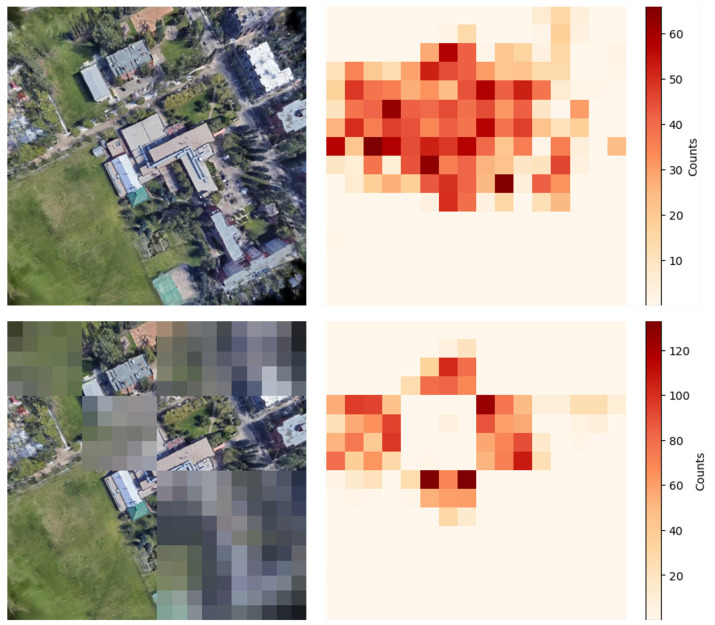
Inliers distributions before and after applying blur and mosaic effects.

Moreover, Figure 18 shows heatmaps that visualize the distribution of inliers for the original and processed rendered images.

As shown in Figure 18, the inliers distribution changed after applying the effects, and the alignment was primarily driven by the unprocessed regions. Therefore, even in natural disasters, alignment may remain feasible by leveraging partially unaffected regions.

## 6. Conclusions

In this study, we aimed to improve image-based geolocalization by generating images from viewpoints close to the satellite viewpoint based on multiview UAV images using 3DGS. Assuming that the approximate flight area of the UAV was known, we directly aligned 3DGS-rendered images with unpartitioned wide-area satellite images using pixel-level image matching. Despite assuming that the UAV yaw angle was unknown, the proposed method achieved higher geolocalization accuracy than those of existing approaches. Furthermore, using pixel-level image matching, we observed a certain degree of robustness to appearance changes and visually similar buildings. We also demonstrated the potential to map multiple 3DGS models onto wide-area satellite images. In the future, we expect that the proposed method can align 3DGS models constructed from post-disaster images with wide-area satellite images acquired before a disaster. This will enable disaster-related digital archives and hazard map construction linked to geographic information.

## Figures and Tables

**Table 1:** Comparison of geolocalization methods.

Method	Query Image	Partitioning of Wide-Area Satellite Image	Accuracy	SDM@1	Time (s)
RoMa	North-facing UAV	×	0.856	0.879	2.22
CAMP	North-facing UAV	✓	0.897	0.927	**1.27**
CAMP Top5 + RoMa	North-facing UAV	✓	0.932	**0.960**	10.22
DINOv2	3DGS-rendered	✓	0.716	0.810	2.33
Proposed method	3DGS-rendered	×	**0.949**	0.950	7.28

**Table 2:** Geolocalization results with different inlier ratio thresholds.

Inlier Ratio Threshold	Accuracy	Time (s)
0.01	0.387	2.11
0.05	0.757	3.20
0.1	0.916	3.94
0.2	0.932	4.70
0.3	0.940	5.13
0.4	0.943	5.31
0.5	0.944	6.48

**Table 3:** Geolocalization results of CAMP for different partition offsets.

Offset (Pixels)	Recall@1	Recall@5
0	0.892	0.961
30	0.793	0.954
50	0.519	0.829

**Table 4:** Geolocalization results with different spatial resolutions (N=100).

Spatial Resolution	Accuracy	SDM@1
0.7 m	0.950	0.955
1.0 m	0.700	0.732

**Table 5:** Geolocalization accuracy versus the proportion of blur and mosaic regions in rendered images.

Number of Blurred Blocks	Blurred Ratio	Accuracy (Mean ± Std)
2	0.125	0.920 ± 0.010
4	0.250	0.882 ± 0.012
6	0.375	0.814 ± 0.005
8	0.500	0.724 ± 0.024
10	0.625	0.573 ± 0.029
12	0.750	0.344 ± 0.044
14	0.875	0.065 ± 0.035

## Data Availability

Restrictions apply to the availability of these data. Part of the data used in this study were obtained from the University-1652 dataset and are available at https://github.com/layumi/University1652-Baseline (accessed on 7 January 2026) with the permission of the University-1652 dataset authors. The remaining original data are not publicly available due to copyright. They are available on request from the corresponding author. The code for the proposed method is publicly available at https://github.com/tus-arakawa/geolocalization (accessed on 5 February 2026).

## References

[1] Wang J., Wang P., Qu L., Pei Z., Ueda T. (2024). Automatic detection of building surface cracks using UAV and deep learning-combined approach. Struct. Concr..

[2] Partama I.G.Y., Yastika P.E., Wijaya I.M.W. (2025). 3D Modeling using UAV-photogrammetry technique for digital documentation of cultural heritage buildings. GEOMATE J..

[3] Obaid L., Hamad K., Al-Ruzouq R., Dabous S.A., Ismail K., Alotaibi E. (2025). State-of-the-art review of unmanned aerial vehicles (UAVs) and artificial intelligence (AI) for traffic and safety analyses: Recent progress, applications, challenges, and opportunities. Transp. Res. Interdiscip. Perspect..

[4] Lin H.Y., Chen L.Y. (2025). GNSS-denied UAV localization with satellite and aerial image matching. Results Eng..

[5] Durgam A., Paheding S., Dhiman V., Devabhaktuni V. (2024). Cross-View Geo-Localization: A Survey. IEEE Access.

[6] Moskalenko I., Kornilova A., Ferrer G. (2025). Visual place recognition for aerial imagery: A survey. Robot. Auton. Syst..

[7] Couturier A., Akhloufi M.A. (2024). A Review on Deep Learning for UAV Absolute Visual Localization. Drones.

[8] Hu S., Feng M., Nguyen R.M.H., Lee G.H. (2018). CVM-Net: Cross-View Matching Network for Image-Based Ground-to-Aerial Geo-Localization. Proceedings of the 2018 IEEE/CVF Conference on Computer Vision and Pattern Recognition, Salt Lake City, UT, USA, 18–23 June 2018.

[9] Keetha N., Mishra A., Karhade J., Jatavallabhula K.M., Scherer S., Krishna M., Garg S. (2024). AnyLoc: Towards Universal Visual Place Recognition. IEEE Robot. Autom. Lett..

[10] Arandjelovic R., Gronat P., Torii A., Pajdla T., Sivic J. (2016). NetVLAD: CNN architecture for weakly supervised place recognition. Proceedings of the IEEE Conference on Computer Vision and Pattern Recognition, Las Vegas, NV, USA, 27–30 June 2016.

[11] Oquab M., Darcet T., Moutakanni T., Vo H.V., Szafraniec M., Khalidov V., Fernandez P., Haziza D., Massa F., El-Nouby A. (2024). DINOv2: Learning Robust Visual Features without Supervision. Trans. Mach. Learn. Res..

[12] Deuser F., Habel K., Oswald N. (2023). Sample4Geo: Hard Negative Sampling For Cross-View Geo-Localisation. Proceedings of the IEEE/CVF International Conference on Computer Vision (ICCV), Paris, France, 1–6 October 2023.

[13] Wu Q., Wan Y., Zheng Z., Zhang Y., Wang G., Zhao Z. (2024). CAMP:A Cross-View Geo-Localization Method Using Contrastive Attributes Mining and Position-Aware Partitioning. IEEE Trans. Geosci. Remote Sens..

[14] Hao Y., He M., Liu Y., Liu J., Meng Z. (2023). Range–Visual–Inertial Odometry with Coarse-to-Fine Image Registration Fusion for UAV Localization. Drones.

[15] Ye Y., Teng X., Chen S., Li Z., Liu L., Yu Q., Tan T. (2025). Exploring the best way for UAV visual localization under Low-altitude Multi-view Observation Condition: A Benchmark. arXiv.

[16] Gurgu M.M., Queralta J.P., Westerlund T. (2022). Vision-Based GNSS-Free Localization for UAVs in the Wild. Proceedings of the 2022 7th International Conference on Mechanical Engineering and Robotics Research (ICMERR), Krakow, Poland, 9–11 December 2022.

[17] Dhaouadi O., Marin R., Meier J., Kaiser J., Cremers D. (2025). OrthoLoC: UAV 6-DoF Localization and Calibration Using Orthographic Geodata. arXiv.

[18] Nassar A., Elhelw M. (2020). Aerial Imagery Registration Using Deep Learning for UAV Geolocalization. Deep Learning in Computer Vision.

[19] He J., Wu Q. (2025). A Localization Method for UAV Aerial Images Based on Semantic Topological Feature Matching. Remote Sens..

[20] Goodfellow I.J., Pouget-Abadie J., Mirza M., Xu B., Warde-Farley D., Ozair S., Courville A., Bengio Y. (2014). Generative adversarial nets. Proceedings of the 28th International Conference on Neural Information Processing Systems, Montreal, QC, Canada, 8–13 December 2014.

[21] Tian X., Shao J., Ouyang D., Shen H.T. (2022). UAV-Satellite View Synthesis for Cross-View Geo-Localization. IEEE Trans. Circuits Syst. Video Technol..

[22] Ho J., Jain A., Abbeel P., Larochelle H., Ranzato M., Hadsell R., Balcan M., Lin H. (2020). Denoising Diffusion Probabilistic Models. Proceedings of the Advances in Neural Information Processing Systems, Online, 6–12 December 2020.

[23] Arrabi A., Zhang X., Sultani W., Chen C., Wshah S. Cross-View Meets Diffusion: Aerial Image Synthesis with Geometry and Text Guidance. Proceedings of the 2025 IEEE/CVF Winter Conference on Applications of Computer Vision (WACV).

[24] Li H., Xu C., Yang W., Mi L., Yu H., Zhang H., Xia G.S. (2025). Unsupervised Multiview UAV Image Geolocalization via Iterative Rendering. IEEE Trans. Geosci. Remote Sens..

[25] Mildenhall B., Srinivasan P.P., Tancik M., Barron J.T., Ramamoorthi R., Ng R. (2021). NeRF: Representing Scenes as Neural Radiance Fields for View Synthesis. Commun. ACM.

[26] Kerbl B., Kopanas G., Leimkuehler T., Drettakis G. (2023). 3D Gaussian Splatting for Real-Time Radiance Field Rendering. ACM Trans. Graph..

[27] Huang Q., Guo X., Wang Y., Sun H., Yang L. (2024). A survey of feature matching methods. IET Image Process..

[28] Lowe D.G. (2004). Distinctive image features from scale-invariant keypoints. Int. J. Comput. Vis..

[29] Bay H., Tuytelaars T., Van Gool L. (2006). Surf: Speeded up robust features. Proceedings of the European Conference on Computer Vision, Graz, Austria, 7–13 May 2006.

[30] Rublee E., Rabaud V., Konolige K., Bradski G. (2011). ORB: An efficient alternative to SIFT or SURF. Proceedings of the 2011 International Conference on Computer Vision, Barcelona, Spain, 6–13 November 2011.

[31] DeTone D., Malisiewicz T., Rabinovich A. (2018). Superpoint: Self-Supervised Interest Point Detection and Description. Proceedings of the IEEE Conference on Computer Vision and Pattern Recognition Workshops, Salt Lake City, UT, USA, 18–22 June 2018.

[32] Tyszkiewicz M., Fua P., Trulls E. (2020). DISK: Learning local features with policy gradient. Adv. Neural Inf. Process. Syst..

[33] Sarlin P.E., DeTone D., Malisiewicz T., Rabinovich A. (2020). SuperGlue: Learning Feature Matching With Graph Neural Networks. Proceedings of the IEEE/CVF Conference on Computer Vision and Pattern Recognition (CVPR), Seattle, WA, USA, 13–19 June 2020.

[34] Lindenberger P., Sarlin P.E., Pollefeys M. (2023). LightGlue: Local Feature Matching at Light Speed. Proceedings of the 2023 IEEE/CVF International Conference on Computer Vision (ICCV), Paris, France, 1–6 October 2023.

[35] Vaswani A., Shazeer N., Parmar N., Uszkoreit J., Jones L., Gomez A.N., Kaiser Ł., Polosukhin I. (2017). Attention is all you need. Adv. Neural Inf. Process. Syst..

[36] Sun J., Shen Z., Wang Y., Bao H., Zhou X. (2021). LoFTR: Detector-Free Local Feature Matching with Transformers. Proceedings of the 2021 IEEE/CVF Conference on Computer Vision and Pattern Recognition (CVPR), Nashville, TN, USA, 20–25 June 2021.

[37] Edstedt J., Sun Q., Bökman G., Wadenbäck M., Felsberg M. (2024). RoMa: Robust Dense Feature Matching. Proceedings of the 2024 IEEE/CVF Conference on Computer Vision and Pattern Recognition (CVPR), Seattle, WA, USA, 16–22 June 2024.

[38] He X., Yu H., Peng S., Tan D., Shen Z., Bao H., Zhou X. (2025). MatchAnything: Universal Cross-Modality Image Matching with Large-Scale Pre-Training. arXiv.

[39] Edstedt J., Nordström D., Zhang Y., Bökman G., Astermark J., Larsson V., Heyden A., Kahl F., Wadenbäck M., Felsberg M. (2025). RoMa v2: Harder Better Faster Denser Feature Matching. arXiv.

[40] Wang S., Leroy V., Cabon Y., Chidlovskii B., Revaud J. (2024). DUSt3R: Geometric 3D Vision Made Easy. Proceedings of the 2024 IEEE/CVF Conference on Computer Vision and Pattern Recognition (CVPR), Seattle, WA, USA, 16–22 June 2024.

[41] Leroy V., Cabon Y., Revaud J. (2024). Grounding Image Matching in 3D with MASt3R. Proceedings of the European Conference on Computer Vision, Milan, Italy, 29 September–4 October 2024.

[42] Yang Y., Xu L., Wang B., Han Y., Ye D. (2025). Absolute Visual Localization for Aerial Vehicles Based on Deep Learning Image Matching. IPMML ’24: Proceedings of the 2024 International Conference on Image Processing, Multimedia Technology and Maching Learning, Dali, China, 27–29 December 2024.

[43] Dai M., Zheng E., Feng Z., Qi L., Zhuang J., Yang W. (2024). Vision-Based UAV Self-Positioning in Low-Altitude Urban Environments. IEEE Trans. Image Process..

[44] Liu G., Li Z., Gao Q., Yuan Y. (2025). SAVL: Scene-Adaptive UAV Visual Localization Using Sparse Feature Extraction and Incremental Descriptor Mapping. Remote Sens..

[45] Jiang P., Bendapudi N., Saripalli S., Pandey G. (2025). 3DGS-Loc: 3D Gaussian Splatting for Map Representation and Visual Localization. J. Auton. Veh. Syst..

[46] Jun H., Yu H., Oh S. (2024). Renderable Street View Map-Based Localization: Leveraging 3D Gaussian Splatting for Street-Level Positioning. Proceedings of the 2024 IEEE/RSJ International Conference on Intelligent Robots and Systems (IROS), Abu Dhabi, United Arab Emirates, 14–18 October 2024.

[47] Radenović F., Tolias G., Chum O. (2019). Fine-Tuning CNN Image Retrieval with No Human Annotation. IEEE Trans. Pattern Anal. Mach. Intell..

[48] Ju H., Huang S., Liu S., Zheng Z. (2025). Video2BEV: Transforming Drone Videos to BEVs for Video-based Geo-localization. Proceedings of the IEEE/CVF International Conference on Computer Vision, Honolulu, HI, USA, 19–25 October 2025.

[49] Zheng Z., Wei Y., Yang Y. (2020). University-1652: A Multi-view Multi-source Benchmark for Drone-based Geo-localization. MM ’20, Proceedings of the 28th ACM International Conference on Multimedia, Seattle, WA, USA, 12–16 October 2020.

[50] Zhu R., Yin L., Yang M., Wu F., Yang Y., Hu W. (2023). SUES-200: A Multi-Height Multi-Scene Cross-View Image Benchmark Across Drone and Satellite. IEEE Trans. Circuits Syst. Video Technol..

[51] Schönberger J.L., Frahm J.M. (2016). Structure-from-Motion Revisited. Proceedings of the 2016 IEEE Conference on Computer Vision and Pattern Recognition (CVPR), Las Vegas, NV, USA, 27–30 June 2016.

[52] Fischler M.A., Bolles R.C. (1981). Random sample consensus: A paradigm for model fitting with applications to image analysis and automated cartography. Commun. ACM.

[53] Fang S., Shen I., Igarashi T., Wang Y., Wang Z., Yang Y., Ding W., Zhou S. (2025). Nerf is a valuable assistant for 3d gaussian splatting. Proceedings of the IEEE/CVF International Conference on Computer Vision, Honolulu, HI, USA, 19–25 October 2025.

[54] Tang J., Gao Y., Yang D., Yan L., Yue Y., Yang Y. (2025). DroneSplat: 3D Gaussian Splatting for Robust 3D Reconstruction from In-the-Wild Drone Imagery. Proceedings of the 2025 IEEE/CVF Conference on Computer Vision and Pattern Recognition (CVPR), Nashville, TN, USA, 10–17 June 2025.

[55] Jiang L., Ren K., Yu M., Xu L., Dong J., Lu T., Zhao F., Lin D., Dai B. (2025). Horizon-GS: Unified 3D Gaussian Splatting for Large-Scale Aerial-To-Ground Scenes. Proceedings of the 2025 IEEE/CVF Conference on Computer Vision and Pattern Recognition (CVPR), Nashville, TN, USA, 10–17 June 2025.

[56] Zhang C., Cao Y., Zhang L. (2025). CrossView-GS: Cross-view Gaussian Splatting For Large-scale Scene Reconstruction. arXiv.

[57] Liu Y., Luo C., Fan L., Wang N., Peng J., Zhang Z. (2024). CityGaussian: Real-Time High-Quality Large-Scale Scene Rendering with Gaussians. Proceedings of the European Conference on Computer Vision, Milan, Italy, 29 September–4 October 2024.

[58] Lin J., Li Z., Tang X., Liu J., Liu S., Liu J., Lu Y., Wu X., Xu S., Yan Y. (2024). VastGaussian: Vast 3D Gaussians for Large Scene Reconstruction. Proceedings of the 2024 IEEE/CVF Conference on Computer Vision and Pattern Recognition (CVPR), Seattle, WA, USA, 16–22 June 2024.

[59] Lee J.Y., Liu Y.R., Tsai S.R., Chang W.C., Wu C.H., Chan J., Zhao Z., Lin C.H., Liu Y.L. (2025). Skyfall-GS: Synthesizing Immersive 3D Urban Scenes from Satellite Imagery. arXiv.

[60] Chen Z. (2024). Application of UAV remote sensing in natural disaster monitoring and early warning: An example of flood and mudslide and earthquake disasters. Highlights Sci. Eng. Technol..

[61] Yucesoy E., Balcik B., Coban E. (2025). The role of drones in disaster response: A literature review of operations research applications. Int. Trans. Oper. Res..

[62] Shibayama A., Kitamura M., Boret P.S., Imamura F. (2018). Higashi nihon daishinsai kara miete kuru shinsai akaibu no genjot o kadai [the state and visions of disaster archives as seen from the Great East Japan earthquake]. Digit. Arch. Gakkaishi.

[63] Hiroki T., Boret S.P. (2021). The Value of Visual Disaster Records from Digital Archives and Films in Post-3/11 Japan. Int. J. Sustain. Future Hum. Secur..

[64] Gerster J., Boret S.P., Morimoto R., Gordon A., Shibayama A. (2022). The potential of disaster digital archives in disaster education: The case of the Japan disasters digital archive (JDA) and its geo-location functions. Int. J. Disaster Risk Reduct..

